# Acetan and Acetan-Like Polysaccharides: Genetics, Biosynthesis, Structure, and Viscoelasticity

**DOI:** 10.3390/polym13050815

**Published:** 2021-03-07

**Authors:** Janja Trček, Iztok Dogsa, Tomaž Accetto, David Stopar

**Affiliations:** 1 Department of Biology, Faculty of Natural Sciences and Mathematics, University of Maribor, SI-2000 Maribor, Slovenia; 2 Faculty of Chemistry and Chemical Engineering, University of Maribor, SI-2000 Maribor, Slovenia; 3 Department of Food Science and Technology, Biotechnical Faculty, University of Ljubljana, SI-1000 Ljubljana, Slovenia; Iztok.Dogsa@bf.uni-lj.si (I.D.); David.Stopar@bf.uni-lj.si (D.S.); 4 Animal Science Department, Biotechnical Faculty, University of Ljubljana, SI-1230 Domžale, Slovenia; Tomaz.Accetto@bf.uni-lj.si

**Keywords:** *Komagataeibacter*, *Acetobacter*, extracellular polysaccharide, acetan, acetan-like polysaccharide, acetan genetic cluster, acetan-like polysaccharide genetic cluster, acetan structure, acetan synthesis, acetan viscoelasticity, applications of acetan

## Abstract

Bacteria produce a variety of multifunctional polysaccharides, including structural, intracellular, and extracellular polysaccharides. They are attractive for the industrial sector due to their natural origin, sustainability, biodegradability, low toxicity, stability, unique viscoelastic properties, stable cost, and supply. When incorporated into different matrices, they may control emulsification, stabilization, crystallization, water release, and encapsulation. Acetan is an important extracellular water-soluble polysaccharide produced mainly by bacterial species of the genera *Komagataeibacter* and *Acetobacter*. Since its original description in *Komagataeibacter xylinus*, acetan-like polysaccharides have also been described in other species of acetic acid bacteria. Our knowledge on chemical composition of different acetan-like polysaccharides, their viscoelasticity, and the genetic basis for their production has expanded during the last years. Here, we review data on acetan biosynthesis, its molecular structure, genetic organization, and mechanical properties. In addition, we have performed an extended bioinformatic analysis on acetan-like polysaccharide genetic clusters in the genomes of *Komagataeibacter* and *Acetobacter* species. The analysis revealed for the first time a second acetan-like polysaccharide genetic cluster, that is widespread in both genera. All species of the *Komagataeibacter* possess at least one acetan genetic cluster, while it is present in only one third of the *Acetobacter* species surveyed.

## 1. Introduction

**Figure 1:**
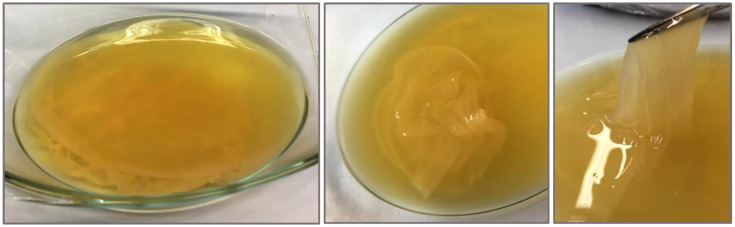
Growing of *Komagataeibacter pomaceti* T5K1^T^ in static culture for production of acetan-containing biofilm.

Acetan is a water-soluble polysaccharide produced by bacterium *Komagataeibacter xylinus* (previously classified as *Gluconacetobacter xylinus* and *Acetobacter xylinum*) [1,2]. After synthesis, it is transported out of the cell, where it is an important constituent of the extracellular matrix of a floating biofilm at the static liquid–air interface (Figure 1). This keeps acetic acid bacteria in a close contact with oxygen that is obligatory for their growth. A substantial amount of acetan is also released into the medium. The biofilm and soluble acetan may have different monosaccharide compositions [3,4].

*K. xylinus* also produces cellulose, an insoluble extracellular polysaccharide that is dispersed better in the presence of acetan. This hinders binding of cells to the cellulose fibers and consequently increases the bacterial growth and cellulose production, especially when grown in shaking culture [5]. The important positive effect of acetan on cellulose production and yield by *K. xylinus* has also been suggested by Sangok et al. [6]. The recent studies additionally showed that acetan modulates bundling of cellulose ribbons and thus alters cellulose formation and assembly [3]. The low pH in the growth medium, tolerated by many *Komagataeibacter* species, reduces the risk for contamination by other microorganisms, but does not reduce the production of extracellular acetan [7,8]. In *Gluconacetobacter diazotrophicus*, a plant-colonizing nitrogen-fixing endophyte, an acetan-like polysaccharide is required for biofilm formation and plant colonization [9,10]. Acetan resembles commercially important polysaccharide xanthan with some distinct features: acetan has pentasaccharide instead of the trisaccharide side group [11], which may change intermolecular interactions and contribute to the formation of more complex supramolecular structures. In contrast to xanthan, which is produced by a phytopathogen *Xanthomonas campestris* [12], the acetan is produced by *Komagataeibacter* and other acetic acid bacteria which belong to GRAS (Generally Recognized As Safe) bacteria.

Our knowledge on the chemical composition of different acetan-like polysaccharides, their viscoelasticity, and the genetic basis for their production has expanded during the last years. Although acetan and acetan-like polysaccharides have attracted attention during the last years, the factors which induce and regulate acetan production have not been studied thoroughly. This is the first review on acetan and acetan-like polysaccharides, and includes analysis of recent genomic data, acetan biosynthesis, its molecular structure, and mechanical properties.

## 2. Chemical Composition and Synthesis of Acetan and Acetan-Like Polysaccharides

**Figure 2:**
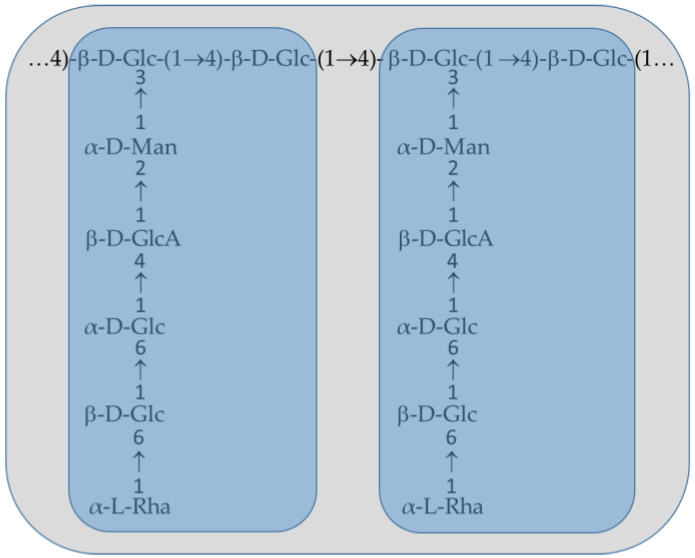
Schematic linkage between two repeat units (framed in blue) in acetan of *Komagataeibacter xylinus*.

The presence of a water-soluble extracellular polysaccharide in a cellulose-negative strain of *K. xylinus,* has been suggested already in 1981 by Valla and Kjosbakken [13], and has been later described and named as acetan by Couso et al. [1,14]. In *Komagataeibacter xylinus* B42, a repeat unit of acetan is composed of α, l-rhamnose-(1,6)-β, d-glucose-(1,6)-α, d-glucose-(1,4)-β, d-glucuronic acid-(1,2)-α, d-mannose-(1,3)-β, d-glucose-(1,4)-β, d-glucose [1,15]. The main chain of acetan is composed of β-1,4-linked d-glucose residues, with every second glucose branched with pentasaccharide [1] (Figure 2). The repeat unit is acetylated at two sites: one at position C6 of the inner mannosyl residue and the other on the polymer backbone, probably at C6 of the branched glucosyl residues [16]. In the genome sequence of *K. xylinus*, there is a homologue to GumF, an acetylase of xanthan in *X. campestris* [17]. The presence of acetyl groups is important for acetan conformation and affects chain–chain interactions, solubility, water-holding capacity, viscoelasticity, and molecular weight [18].

**Figure 3:**
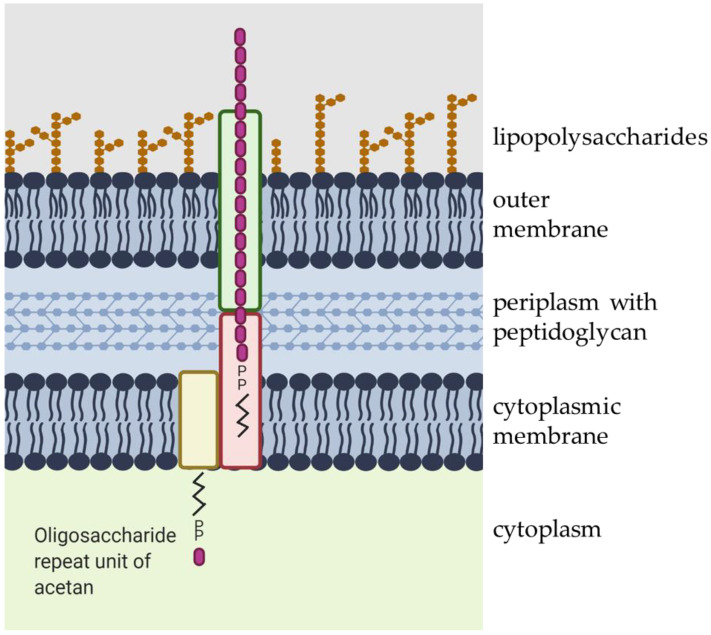
Schematic presentation of acetan secretion. The acetan oligosaccharide repeat unit is transported across the cytoplasmic membrane, assembled in periplasm, and transported out of the cell. The figure was created with Biorender.com.

The acetan repeat unit is synthesized by sequential addition of individual sugar units to the activated lipid-phosphate by specific glycosyl transferases in the cytoplasm, followed by polymerization in the periplasm, and secretion outside of the cell [19] (Figure 3). The biochemical pathway for acetan production has been studied in *K. xylinus* using a cell-free extract and radiolabeled sugar nucleotide precursors [20,21]. So far, only a few enzymes have been purified and characterized [22]. The function of enzymes involved in acetan production has been mainly deduced from the genome sequence of *K. xylinus* after comparing proteins involved in the putative acetan synthesis to proteins involved in production of other water-soluble heteropolysaccharides, in particular xanthan [23].

**Figure 4:**
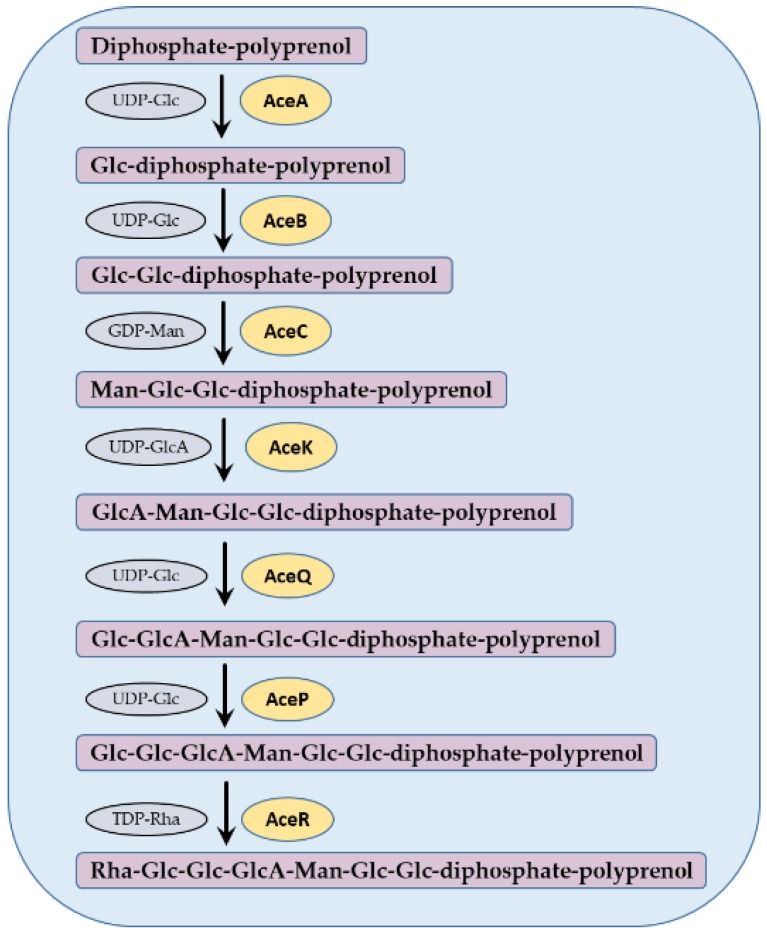
Biochemical steps in acetan repeat unit synthesis by *Komagataeibacter xylinus*. In each step, an addition of a specific activated monosaccharide (in gray) to a growing intermediate chain of acetan repeat unit is catalyzed by a specific enzyme (in yellow).

The acetan biosynthesis proceeds in the following seven biochemical reaction steps (Figure 4). In the initial step, a glucosyl-1-phosphate is transferred from uridine diphosphate-glucose (UDP-Glc) to diphosphate-polyprenol lipid, resulting in glucose-diphospho-polyprenol. This reaction is catalyzed by AceA in *K. xylinus* [24]. The next enzyme, AceB, adds a second glucosyl residue from UDP-Glc to Glc-diphospho-polyprenol, resulting in cellobiosyl-diphospho-polyprenol [24,25]. The disaccharide is enlarged by adding a mannosyl residue from guanosine diphospate-mannose (GDP-Man) by mannosyl transferase enzyme denoted AceC [26]. In the next step, a glucuronyl residue is added from UDP-glucuronate (UDP-GlcA) to a lipid-linked Glc-Glc-Man trisaccharide. This putative enzyme has a high homology to GumK [23], therefore we named it AceK in Figure 4. The next enzyme, AceQ, is a glucosyl transferase, which transfers a glucose residue from UDP-Glc to GlcA-Man-Glc-Glc-diphospho-polyprenol [22]. Another glucose residue is added by AceP, a glucosyl transferase which transfers a Glc from UDP-Glc, resulting in Glc-Glc-GlcA-Man-Glc-Glc-diphospho-polyprenol [27]. The final sugar residue is added by AceR, a rhamnosyl transferase, which transfers a rhamnosyl residue from thymidine-rhamnose (TDP-Rha), resulting in Rha-Glc-GlcA-Glc-Glc-Man-Glc-Glc-diphospho-polyprenol [22] (Figure 4).

After synthesis, lipid-linked heptasaccharide repeating units are polymerized in a sequential series of reactions and transported out of the cell [14]. The polymerization and export of acetan in the *K. xylinus* seems to follow the Wzx/Wzy-dependent pathway due to the presence of *aceD*, *aceE*, *aceG*, and *aceH* genes, which putatively code for polysaccharide copolymerase, flippase, beta-barrel porin, and outer membrane transport protein, respectively [24,28].

The presence of acetan-like extracellular water-soluble polysaccharides has been studied and partially characterized also in *Komagataeibacter hansenii*, *Komagataeibacter sucrofermentans*, *Acetobacter estunensis*, *Gluconacetobacter diazotrophicus,* and *Kozakia baliensis*. The monosaccharide composition of these extracellular water-soluble acetan-like polysaccharides are presented in Table 1. Most of the data are for different *Komagataeibacter* species. Glucose and mannose are present in all acetan-like polysaccharides, and the other monosaccharides differ among species and strains. Data in Table 1 also show that the monosaccharide composition of polysaccharides may differ if material is harvested directly by precipitation from the growth medium or extracted from the cellulolytic biofilm. The acetan-like polysaccharide from *Gluconacetobacter diazotrophicus* Pal5 is composed of glucose, galactose, and mannose in molar ratio of 6:3:1. In contrast to acetan, this polysaccharide does not contain glucuronic acid and has the majority of monosaccharides in β-anomeric configuration, which probably contributes to its poor solubility in water [10]. The polysaccharide of *Kozakia baliensis* NBRC 16680 is composed of glucose, mannose, glucuronic or uronic acid, galactose, or glucose, and it is not acetylated in contrast to acetan [29].

As shown in Table 1, the molecular weights of acetan-like polysaccharides differ. This may be attributed to different types of acetan-like polysaccharides, but also to different methods used for polysaccharides’ purification. Typically, the acetan-like polysaccharides are purified from the supernatant by ethanol-precipitation in the presence of KCl or are extracted from biofilm by stepwise separation of cells, proteins, and cellulose using NaOH, centrifugation, and dialysis [3]. The authors used different variations of protocols, such as disruption of biofilm in a blender and filtration through several layers of cheesecloth [1], filtration through filters with defined pore size [13], suspensions of proteins in different types and concentrations of salts before polysaccharide precipitation [1,13,34], extraction of proteins by phenol [13], precipitation of polysaccharides by different alcohols or cetytrimethyl ammonium bromide [1,34], as well as separation of precipitate at different centrifugal speeds [1,13,30,32]. Moreover, differences may also be a result of different separation columns and protocols used in size exclusion chromatography on a high performance liquid chromatography system [29,33].

It is known that the carbon source may influence monosaccharide composition of water-soluble heteropolysaccharides [35]. However, Minakami et al. [34] reported that *A. estunensis* produced the same monosaccharide structure of the water-soluble polysaccharide on different carbon sources. Kornmann et al. [32] reported that nutritional factors had a strong influence on the yield of acetan produced by *K. xylinus*, however, its molecular composition was independent of the production conditions. In contrast, the results of Fang and Catchmark [3] suggest that carbon source influenced the monosaccharide composition of extracellular water-soluble polysaccharide in some *Komagataeibacter* strains. Recently, Brandt et al. [29] reported that acetan-like polysaccharides from *K. baliensis*, which were produced in media with different carbon sources, showed the same monomer composition, but varying rheological and macromolecular properties, which was attributed to different acetylation of polysaccharide.

Knowledge about higher molecular structures of extracellular water-soluble heteropolysaccharides produced by *Komagataeibacter* spp. and *Acetobacter* spp. is poor. From the two genera, only acetan of *K. xylinus* B42 has been studied in some detail [1,11,16,36]. The nuclear magnetic resonance (NMR) analysis of the native, deacetylated, and enzymatically depolymerized acetan revealed the primary structure and connectivity between monosaccharides of acetan, as presented in Figure 2.

## 3. Genetic Basis of Acetan Production in *Komagataeibacter* spp. and *Acetobacter* spp.

The pioneering studies pinpointing the genetic locus coding for enzymes and transporters involved in *K. xylinus* acetan production go back to the 1990s. Then, several genes of this biosynthesis cluster were isolated and sequenced using the classical cloning approaches [15,37,38]. The functional annotation relied heavily on the observed homology to the better known xanthan biosynthesis gene cluster of *X. campestris* [39,40]. Some functional studies were also done by construction of specific *K. xylinus* mutants [22], identification of appropriate enzymatic activities in *Escherichia coli* strains transformed with acetan cluster genes [15], and complementation of xanthan-deficient mutants of *X. campestris* [26]. In recent years, *K. baliensis* acetan-like polysaccharide and its biosynthesis locus were characterized [25,29]. Although, the *K. baliensis* locus had quite similar architecture and high amino acid sequence similarity of coded products to those of *K. xylinus*, the differences in acetan monosaccharide composition, such as the presence of rhamnose in *K. xylinus* acetan, were linked to the presence of extra genes in the latter locus. A survey of several *Komagataeibacter* species identified proteins homologous to the majority of those coded in *K. xylinus* acetan cluster, also in species *K. europaeus*, *K. intermedius*, *K. oboediens*, *K. medellinensis,* and *K. rhaeticus*, while *K. hansenii* strains harbored only homologues involved in acetan polymerization and export [28].

Here, we extended bioinformatic studies to the newly described and sequenced *Komagataeibacter* species and strains using the *K. xylinus* E25 cluster as a starting protein set. The set included proteins involved in acetan synthesis initiation, polymerization, and export (AceA, AceD, AceE, AceG, AceH, GumE), glycosyl transferases (AceB, AceC, AceP, AceQ, AceR, GumK), acetyl/acyl transferases (GumF, AceI), enzymes involved in synthesis of nucleotide sugars (AceF, AceM), and a glycoside hydrolase (Eg). When searching for homologues in *Komagataeibacter*, we noticed that this biosynthetic locus was conserved only in one part of the genus (Supplementary Table S1), namely in species forming a well-resolved phylogenetic cluster around *K. xylinus* as identified by rRNA phylogeny reconstruction [2]. However, the lack the whole locus was confirmed in strain *K. europaeus* LMG 18494. The locus also seemed unstable in species *K. oboediens*, where strain LMG 18849 did not have the locus, and in strain 174Bp2, the critical *aceA* gene was disrupted by an insertion sequence.

When *K. hansenii* strains were further investigated, it became obvious that they possess homologues to proteins involved in acetan synthesis but with profoundly lower similarities compared to species with full acetan loci [28]. As noted by Ryngajłło et al. [28], the vicinity of these low-homology regions contained predicted glycosyl and acyl transferases. We next looked for this kind of region across the genus and found that it is conserved in *K. hansenii* lineage, but completely absent in the *K. xylinus* lineage. It is composed of twelve genes (Supplementary Table S1), and all but one had Genbank annotations in agreement with those of the already known *K. xylinus* acetan locus. We next checked for Pfam [41] and CAZy [42] domains and confirmed that the region indeed harbors the necessary genes enabling the initiation of polysaccharide chain growth, polymerization and its export (corresponding to *aceA*, *aceD*, *aceE*, *aceG*, and *aceH*), four glycosyl transferases, acyl transferase, and a nucleotide-sugar epimerase. There are two glycosyl transferases missing in this locus, but one glycosyl transferase (e.g., HNH97_RS06460 in *G. entanii*) harbors two different domains and may be thus a bifunctional protein. On the other end, the glucuronosyl transferase of the CAZy family GT70 is missing, which suggests that this sugar monomer may be missing from polysaccharide in strains containing this second biosynthetic locus for acetan-like polysaccharide production in *Komagataeibacter*. AceH is the most conserved protein in acetan cluster of the *K. xylinus* and in the new acetan locus, with 38–47% amino acid identity over the entire length. The AceA is similarly conserved, but only in one of its domains. As noted also for *K. xylinus* archetype acetan locus, some type species occasionally lacked the entire newly described acetan locus, although it was present in other strains of the species (e.g., *K. saccharivorans* LMG 1582). It remains to be seen whether this was a sequencing/assembly artefact. Also in this acetan locus, a transposase disruption of *aceA* homologue was observed, e.g. in *K. maltaceti* LMG 1529, suggesting that this may indeed be a somewhat unstable trait in *Komagataeibacter*.

The phylogeny of the conserved protein AceH [43] across *Komagataeibacter* genus was further studied (Supplementary Figure S1A). In most of the species, the AceH proteins formed clusters in agreement with the species affiliation, suggesting a vertical inheritance. However, there was an exception: the AceH proteins of the *K. xylinus* strains were scattered throughout the dendrogram, indicating heterogeneity of AceH in this species. To investigate this putative lateral gene transfer event further, we focused only on the archetypal *K. xylinus* acetan locus and reconstructed the phylogenies of its 17 genes for 16 *Komagataeibacter* strains harboring it. All trees were very similar to the *aceH* based tree (Supplementary Figure S1B), which was also confirmed by a super network analysis of these trees [44] (Supplementary Figure S1C). This suggests that the whole acetan cluster may have been transferred to *K. xylinus* on several occasions from a separate *Komagataeibacter* donor.

We have also analyzed the occurrence of both acetan biosynthetic loci in the genus *Acetobacter*. Initially, only type strains of the species were analyzed. If they were negative for both loci, a strain of a given species with the largest and most complete genome was also analyzed. Out of 32 *Acetobacter* species surveyed, 8 contained *K. xylinus* acetan locus homologues, while the newly described acetan-like biosynthetic locus was present in 10 species (Supplementary Table S1). Interestingly, in all but two species, both loci were present. Some loci were fragmented with gene dispersed to at least three genomic positions. The core *aceA–aceH* genes were always found together, with most of the fragmentation occurring in the *eg-aceP* part. The *aceR* gene (coding for rhamnosyl transferase) was universally lost in these loci, and in most species *gumF* (coding for acetyl transferase), *aceI* (coding for acyl transferase), and *aceE* (coding for flippase) genes were missing. The *gumK* and *aceQ* genes coding for glucuronosyl and glucosyl transferases, respectively, were fused in six *Acetobacter* species and possessed both the GT70 and GT4 domains. The newly found acetan-like locus was slightly less fragmented (Supplementary Table S1) and the putative acyl transferase gene of this locus was lost in *Acetobacter*. The two-domain glycosyl transferase gene (containing GT2 and GT4) of this locus was divided into two genes in several species.

**Figure 5:**
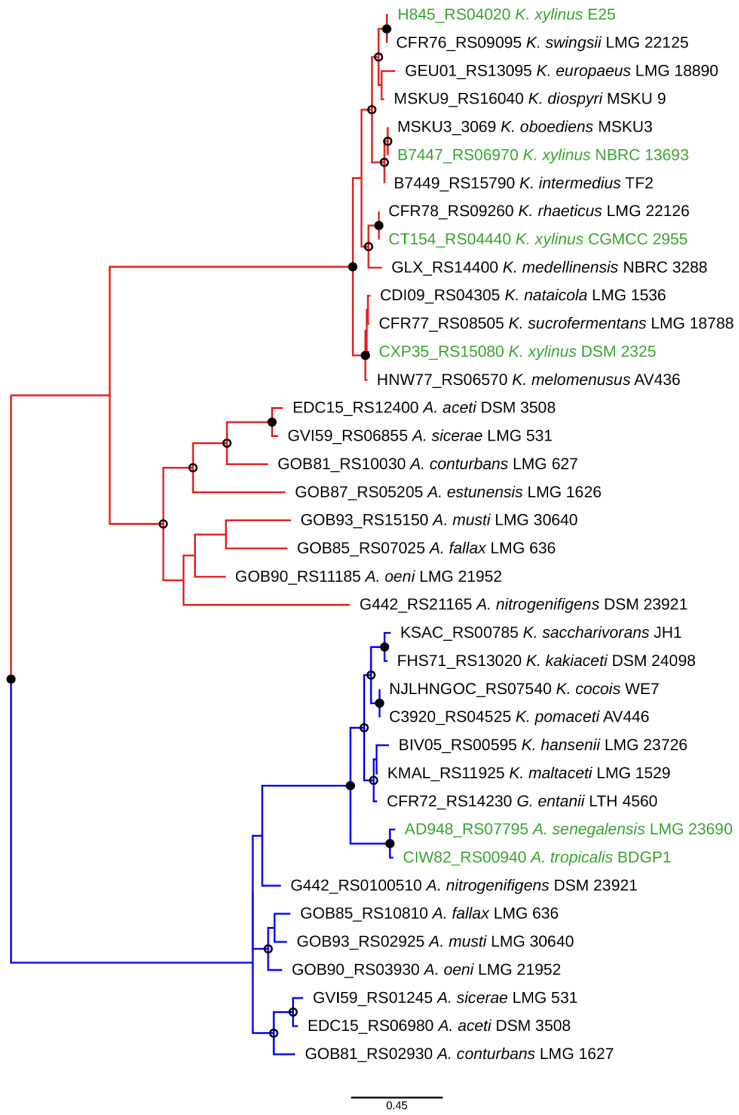
AceH phylogeny reconstruction showing the divergence of the two acetan loci in *Komagataeibacter* and *Acetobacter* as deduced by PhyML using an LG substitution table and 1000 bootstrap replicates. The archetypal *K. xylinus* acetan locus and the newly found locus branches are colored in red and blue, respectively. The strains where acetan locus lateral transfer events may have occurred are in green color. Open and closed node circles indicate bootstrap values above 50% and 90%, respectively. For each AceH, its gene locus tag is also given. The tree was drawn using Iroki [45].

In Figure 5, we present the phylogenetic reconstruction based on AceH of *Komagataeibacter* and *Acetobacter* type strains, and several strains of *K. xylinus*. Clear clustering of AceH belonging to two different acetan-like loci can be seen, as well as a further separation in each of them corresponding to species affiliations. Notably, the *Acetobacter senegalensis* and *Acetobacter tropicalis* AceH proteins cluster with *Komagataeibacter* strains and thus may have been acquired from the *Komagataeibacter* donors. Reinforcing this notion are the facts that the loci of these two species are the only ones in the whole genus, which are not fragmented and additionally not accompanied by another locus, similar to the one in *K. xylinus* E25. Therefore, lateral mobility of acetan biosynthetic clusters in *Acetobacteraceae* evolution may be significant, highlighting their importance in adaptation.

## 4. Viscoelasticity of Acetan Solutions

Acetan is a branched acidic heteropolysaccharide with a profound effect on viscosity. Aqueous solutions of acetan have shear-thinning behavior [46]. This means that viscosity is high at low shear rates and decreases upon shearing. Such a behavior is a direct consequence of acetan solution structure. Optical rotation and circular dichroism studies suggest helical structure in a solution and a helix-coil transition upon heating and cooling. It was concluded from the light-scattering studies that acetan in aqueous solutions exists as a semi-flexible polymer predominantly in double-strand conformation with the persistence length of 100 nm [47,48,49]. The polyelectrolytic nature enables acetan to expand, which can be seen by high Mark-Houwink exponent (a = 0.9, [49]) and intrinsic viscosity, [η], of 8 dl/g (at 0.1 M NaCl), which is typical for swelling polymers. The polymer overlap concentration, where polymer chains start to significantly overlap, is at 1/[η] ≈ 0.125%, which suggests that the observed shear thinning at 0.5% acetan concentration is mostly due to the polymer intermolecular interactions. Consistently, the viscoelastic behavior of acetan with pronounced elasticity was observed in the frequency sweep tests. The storage (G’) and loss (G”) moduli of 0.5% acetan isolated from *Acetobacter xylinum* (presently classified as *Komagataeibacter xylinus*) NNRL B42 suggest that acetan solutions behave as viscoelastic polymer liquids with a crossover point G’ = G” below 3 Hz [46]. The oscillatory frequency data for acetan solutions were characteristic of an entangled polymer solution. The short-ranged entanglements increased if the concentration of acetan was increased to 3%, where weak gel behavior (G’ > G”) was observed with G’ = 33 Pa and G” = 17 Pa at 1 Hz [50].

It has been demonstrated that acetan viscous behavior may depend on the biosynthesis pathway. When *K. baliensis* strains (DSM 14400, NBRC 16680), known to produce large amounts of a soluble acetan-like heteropolysaccharides, were grown in different growth media, the flow behavior of acetan-like polymer differed [29]. For instance, when acetan-like polymer was produced with bacteria grown on mannitol, it displayed less shear-thinning compared to acetan produced on fructose-glucose media supplemented with magnesium. The effect was present in all polymer concentrations tested and was explained with the increased size of the polymer that was attributed to the greater magnesium content in the glucose-fructose medium [29].

Acetan is negatively charged and the effect of salt on viscosity can be significant. For example, a 0.4% aqueous solution of CR1/4 has a viscosity of 4.6 Pa (at a shear rate of 15 s^−1^) compared to 11 Pa in the presence of 0.1 M NaCl. Creep tests at low applied stress also suggest the presence of a small elastic component, indicating structuring of the sample upon salt addition [51]. One would expect that adding high-valence ions might induce gelation in acetan. However, the addition of Cr^3+^ to native acetan did not induce gelling [52].

Acetan solutions exhibit similar rheological properties to xanthan solutions. Besides acetans having pentasaccahrides as side chains, and xanthan trisaccahrides, the two polymers have further important differences. Both acetan and xanthan contain non-carbohydrate substituents (i.e., acetyl groups). Acetan, however, does not contain acetyl or pyruvate substitution at the terminal unit of side chain, but it is partially acetylated along the backbone [53]. Complete deacylation of acetan can be achieved by alkali treatment, heating the aqueous dispersion to 90 °C, cooling it to 1 °C, and then titrating it to pH 12.5 for 16 h [16]. Deacetylation can have a major effect on acetan and xanthan rheological behavior. While deacetylation of xanthan lowers the helix-coil transition temperature, thus destabilizing the helix, the deacetylation of acetan slightly raises the helix-coil transition temperature. The native and completely deacylated acetan have different shear-thinning behavior. The major differences are seen at low shear rates, where de-esterification significantly reduces acetan viscosity [53]. Similar rheological behavior for the related polysaccharide xanthan was explained as a reduced propensity for the intermolecular association. Thus, for acetan, it seems reasonable to suggest that complete de-esterification reduces intermolecular associations.

The effect of the partial removal of acetan side chains was studied in a mutant of *K. xylinus* NRRL B42 that produces acetan CR1/4, which is deficient in the side-chain sugar residues. The CR1/4 acetan has been shown to possess a tetrasaccharide repeat unit with the side chain containing one mannose unit and the terminal unit of glucuronic acid. The X-ray diffraction studies of oriented fibers confirmed that solid-state CR1/4 forms a fivefold helix with a pitch of 4.8 nm, similar to acetylated acetan [46]. Light-scattering studies on CR1/4 solutions suggest a molecular weight of 1.2 × 10^6^ with radii of gyration of 86 nm (in aqueous solution) and 67 nm (in 0.1 M NaCI solution), which is comparable to the native acetan. However, compared to the native acetan, 0.5% solutions of CR1/4 had significantly increased shear-thinning and viscosity. The difference in viscosity was especially prominent at low shear rates and almost completely disappeared at high shear rates [51]. This indicates that decreasing the length and composition of the acetan side chain can be attributed to changes in the polymer–polymer interaction, resulting in increased solution viscosity.

### Viscoelasticity of Acetan Binary Mixtures

Information about acetan interactions with other polymers is limited. It has been shown that acetans can have synergistic interactions with carob (LBG) or konjac mannan. In dilute solutions, the synergistic interactions manifest themselves as increased viscosity of the mixture. At higher concentrations, the mixtures may even form thermo-reversible gels. For the native acetan, the interaction with konjac mannan is much weaker than with carob [54].

It is interesting to note that preliminary studies on dilute mixtures of the native acetan with carob or konjac mannan failed to detect synergistic interactions when samples were mixed, stored, and measured at room temperature [54]. The native acetan and carob mixtures (1% *w*/*v*) gave mechanical spectra approaching those of normal polysaccharide semi-diluted solutions, and there was no evidence of gel formation beyond those of acetan alone. This is consistent with the absence of any detectable enhancement in dilute-solution viscosity [16]. It was also reported that heating the mixtures (up to 60 °C) and cooling to room temperature did not lead to detectable synergistic effects. However, it has been shown that these samples have an excess of NaCl, which may have raised the value of acetan helix-coil transition temperature. Thus, the mixtures may not have been heated to a sufficiently high temperature to enhance the synergistic interaction. Indeed, the synergistic interactions of the native acetan with carob and konjac mannan have been reported when the mixtures were heated to 90 °C, well-above the reported helix-coil transition temperature [54]. The native acetan–carob gels show a maximum in the viscoelastic moduli at a polymer ratio of acetan:carob of 1:1. This is similar to the behavior observed for xanthan–carob mixtures, although the viscoelastic moduli were considerably lower for the acetan mixed gels. The interaction between the native acetan and konjac mannan is much weaker. At 0.5% (w/v), gelation is observed, but the gels are weak, with a maximum viscoelastic moduli at an acetan–konjac mannan ratio of 7:1.

Deacetylation of acetan dramatically enhances the synergism and gel formation with carob or konjac mannans mixtures [16,53,54]. The effect of acetan deacylation parallels the effect of deacylation of xanthan/carob or konjac mannans mixtures. Though, it should be noted that deacylation of xanthan produces much stronger gels. The differences in gel stiffness are most likely due to different effects of xanthan or acetan structure on the formation of gel heterotypic junction zones. The principal differences between xanthan and acetan are the charge density, the mass per unit length, the length and composition of the side chain, and the acetylation of the acetan backbone. It has been shown for completely deacetylated acetan/carob mixtures that gels are stiffer at all polymer ratios and the maximum viscoelastic moduli occur at the acetan:carob ratio of 3:7, instead of 1:1, as seen for the native acetan mixed gels. The binary mixture (3:7) shows typically gel-like behavior, with G’ being substantially higher than G”, and with little variation across the measured frequencies. There was also a large increase in the absolute values of the rheological parameters compared to the starting polymer solutions (e.g., about a 1000-fold increase in a complex viscosity at the lowest accessible frequency (0.1 rad s^−l^)). The effect of acetan de-esterification is even more pronounced on acetan/konjac mannan mixtures. There is a substantial increase in gel stiffness and the maximum modulus value occurs at an acetan–konjac mannan ratio of 6:4 rather than 7:1, as seen for the native acetan mixtures [55].

## 5. Potential Applications of Acetan in Industry

Heteropolysaccharides, such as acetan, have unique properties, since their complex, mostly branched structures are responsible for significant viscosity increase of aqueous solutions. The food industry is taking advantage of the unique rheological properties of bacterial heteropolysaccharides. Nowadays, consumers are increasingly aware of the importance of eating healthy food and the number of consumers who regularly buy food that contains natural ingredients is growing rapidly. Although additives in foods have a bad connotation among consumers, they are indispensable constituents to keep the food matrices microbiologically, chemically, and physically stable. The consumers demand natural additives as an alternative to synthetic additives [56]. The polymers which dissolve or disperse in aqueous solutions and have thickening or gelling properties such as acetan are highly demanded for many industrial applications in food and pharmaceutical industries. In addition, they may cause emulsification, stabilization, suspension, control of crystallization, inhibition of release of water from foods, encapsulation, and film formation [56]. These functional properties are often determined by small differences in polymer structure. The bio-thickeners of bacterial origin are also highly demanded by the medical or pharmaceutical sectors for encapsulation of drugs or probiotics and controlled released of different therapeutic proteins and other formulations [57,58,59].

Bacterial polysaccharides may also have beneficial effects on human health by inferring diverse effects on cells, such as anti-mutagenic, anti-oxidative, and anti-inflammatory effects. This has been extensively studied in lactic acid bacteria [60]. Only limited data exist for acetan AC-1 purified from *Komagataeibacter xylinus* NBI 1005 [31]. Acetan AC-1 induces production of interleukin-12 p40 and tumor necrosis factor-α by macrophages and has in vitro effect by regulating Th2-mediated allergic response. For this reason, it was suggested that AC-1 may be useful in preventing allergy response [61]. Further studies showed that AC-1 after oral administration to mice enhanced the protective immunity against *Listeria monocytogenes* [62] and augmented antitumor activity against different tumors [63].

**Figure 6:**
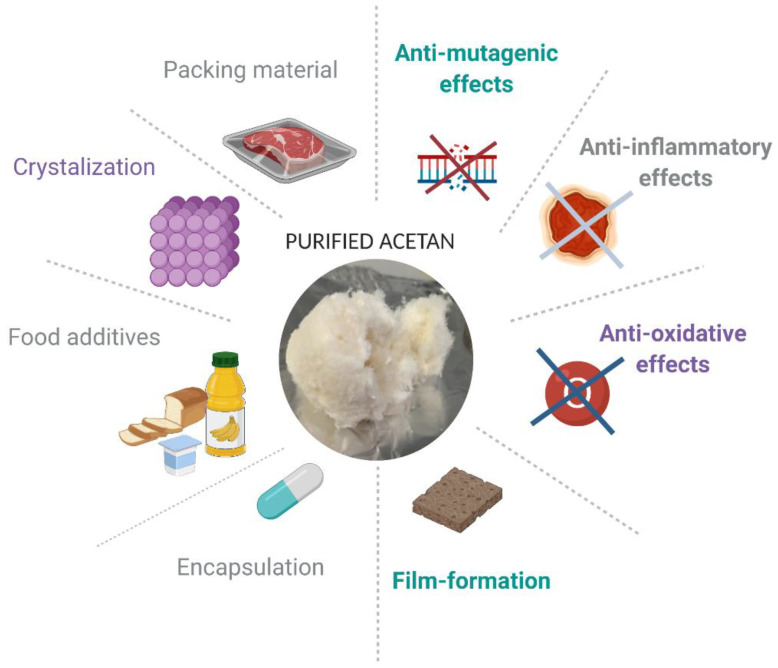
Potential applications of acetan-like polysaccharides. The figure was created with Biorender.com.

Currently, the list of acetan applications is short. We are, however, convinced that acetan and acetan-like polymers have a good potential for commercial use. We see potential applications of acetan in food and pharmaceutical industries (Figure 6). To be as useful as related xanthan polymers, there are several hurdles that need to be overcome prior to commercial applications. We should have a better understanding of its synthesis, composition, and interactions.

## 6. Conclusions and Future Perspectives

As we are getting new genetic information, it becomes obvious that there are several pathways for acetan biosynthesis. Acetan is a generic name for a group of heteropolysaccharides, and the molecular composition of acetan appears to be quite heterogenous across bacterial species and strains. Future in-depth characterization of acetan-like polysaccharides by NMR-spectroscopy in combination with computational methods will establish a database of different types of acetan-like polysaccharides with defined physico-chemical characteristics. Data on structural organization of polysaccharides in different media are scarce but important for acetan modification and preparation of biomaterial with desired characteristics. Relatively little is also known about the size distribution of different acetans in solutions. How different molecular compositions and sizes affect acetan’s physico-chemical properties is largely unknown. Currently, there are only few studies where interaction of acetan with other polymers were characterized. This knowledge is crucial for a wider application of acetan in different matrices important in food and pharmaceutical industries. From a biotechnological point of view, the production yield and purification of acetan should be improved. Despite obvious deficiencies in our current understanding, acetan and acetan-like polymers should be treated as a promising new group of biopolymers with a good potential for applications.

## Figures and Tables

**Table 1:** The monosaccharide composition of acetan-like polysaccharides from different acetic acid bacteria.

Bacterial Strain Designation according to the Present Taxonomy	Carbon Source in Growth Medium	Source of Extracellular Polysaccharide	Molecular Weight of Polysaccharide (Da or g/mol)	Monosaccharide Composition of Water-Soluble Polysaccharide	Analytical Method for Monosaccharides Identification	Reference
*Komagataeibacter xylinus* ATCC 10245, cellulose neg. mutant	Glucose	Precipitation from liquid medium	>10^6^	Glucose, mannose, rhamnose, glucuronic acid	Gas-liquid chromatography, colorimetric analysis	[13]
*Komagataeibacter medellinensis* IFO 3288	Glucose	Precipitation from liquid medium	>1.5 × 10^5^	Glucose, mannose, rhamnose, uronic acid	Gas-liquid chromatography, colorimetric analysis	[30]
*Komagataeibacter xylinus* IFO 13693	Glucose	Precipitation from liquid medium	>1.5 × 10^5^	Glucose, mannose, rhamnose, uronic acid	Gas-liquid chromatography, colorimetric analysis	[30]
*Komagataeibacter xylinus* NBI 1005	Sucrose	Precipitation from liquid medium	10^6^	Glucose, mannose, galactose, glucuronic acid	Paper chromatography, ^13^C-NMR spectroscopy	[31]
*Komagataeibacter xylinus* NRRL B42	Glucose	Extraction from cellulose-biofilm and precipitation from liquid medium	2 × 10^6^	Glucose, mannose, rhamnose, glucuronic acid	Gas-liquid chromatography with mass spectrometry	[1]
*Komagataeibacter xylinus* I-2281	Sucrose or glucose	Precipitation from liquid medium	Not known	Glucose, mannose, rhamnose, glucuronic acid	Anion-exchange chromatography with pulsed amperometric detector	[32]
*Komagataeibacter hansenii* LMG 1524	Sucrose	Precipitation from liquid medium	1.8 × 10^3^, 2.5 × 10^3^	Glucose, galactose, mannose, xylose, arabinose, rhamnose	Gas-chromatography	[33]
*Komagataeibacter hansenii* ATCC 53582	Glucose	Extraction from cellulose-biofilm	Approximately 1.5 × 10^5^, 3 × 10^4^, <5 × 10^3^	Glucose, mannose	Capillary ion chromatography	[3]
*Komagataeibacter hansenii* ATCC 53582	Glucose	Precipitation from liquid medium	Approximately 1.5 × 10^5^, 4 × 10^4^, 10^4^	Glucose, mannose, rhamnose, glucuronic acid	Capillary ion chromatography	[3]
*Komagataeibacter xylinus* ATCC 53524	Glucose	Extraction from cellulose-biofilm	Not known	Glucose, mannose, rhamnose	Capillary ion chromatography	[4]
*Komagataeibacter xylinus* ATCC 53524	Galactose	Extraction from cellulose-biofilm	Not known	Glucose, mannose, rhamnose	Capillary ion chromatography	[4]
*Komagataeibacter xylinus* ATCC 53524	Glucose	Precipitation from liquid medium	Not known	Glucose, mannose, rhamnose, galactose, glucuronic acid	Capillary ion chromatography	[4]
*Komagataeibacter xylinus* ATCC 53524	Galactose	Precipitation from liquid medium	Not known	Glucose, mannose, rhamnose	Capillary ion chromatography	[4]
*Komagataeibacter hansenii* ATCC 53582	Glucose	Extraction from cellulose-biofilm	Not known	Glucose, mannose	Capillary ion chromatography	[4]
*Komagataeibacter hansenii* ATCC 53582	Galactose	Extraction from cellulose-biofilm	Not known	Glucose, mannose, rhamnose	Capillary ion chromatography	[4]
*Komagataeibacter hansenii* ATCC 53582	Glucose	Precipitation from liquid medium	Not known	Glucose, mannose, rhamnose, glucuronic acid	Capillary ion chromatography	[4]
*Komagataeibacter hansenii* ATCC 53582	Galactose	Precipitation from liquid medium	Not known	Glucose, mannose, rhamnose, galactose, glucuronic acid	Capillary ion chromatography	[4]
*Komagataeibacter sucrofermentans* ATCC 700178	Glucose	Extraction from cellulose-biofilm	Not known	Glucose, mannose, rhamnose	Capillary ion chromatography	[4]
*Komagataeibacter sucrofermentans* ATCC 700178	Galactose	Extraction from cellulose-biofilm	Not known	Glucose, mannose	Capillary ion chromatography	[4]
*Komagataeibacter sucrofermentans* ATCC 700178	Glucose	Precipitation from liquid medium	Not known	Glucose, mannose, rhamnose, galactose, glucuronic acid	Capillary ion chromatography	[4]
*Komagataeibacter sucrofermentans* ATCC 700178	Galactose	Precipitation from liquid medium	Not known	Glucose, mannose, rhamnose, galactose, glucuronic acid	Capillary ion chromatography	[4]
*Acetobacter estunensis* IFO 13751^T^	Sucrose	Precipitation from liquid medium	10^6^	Glucose, mannose, galactose, glucuronic acid	Gas-liquid chromatography	[34]
*Gluconacetobacter diazotrophicus* PAI5	Mannitol	Precipitation from liquid medium	8.72 × 10^5^	Glucose, mannose, galactose	Gas chromatography with mass spectroscopy	[10]
*Kozakia baliensis* NBRC 16680	Glucose or fructose and glucose	Precipitation from liquid medium	Not known	Glucose, mannose, glucuronic or uronic acid, galactose or glucose	Gas chromatography with mass spectroscopy, NMR spectroscopy	[29]

## Data Availability

The data presented in this study are available on request from the corresponding author.

## Supplementary Materials

The following are available online at https://www.mdpi.com/2073-4360/13/5/815/s1. Figure S1: Divergence and putative horizontal gene transfer of acetan-like loci in *Komagataeibacter*. A: phylogeny of *Komagataeibacter* AceH as deduced by PhyML using LG substitution table and 1000 bootstrap replicates. The lower cluster corresponds to the archetypal *K. xylinus* E25 locus, while the upper groups show the AceH belonging to the newly found locus. B: *aceH* gene phylogeny of *K. xylinus* E25-type acetan locus reconstructed using GTR model and 1000 bootstrap replicates. *K. xylinus* loci are dispersed among several well-resolved clusters. C: Super-network analysis of 17 gene trees of the 16 *Komagataeibacter* strains harboring the archetypal *K. xylinus* locus. All the trees are essentially very similar, implying the locus was transferred as a unit several times. Table S1: Acetan-like genetic clusters of *Komagataeibacter* and *Acetobacter* species. *K. xylinus* E25 locus was used as a reference.

## Abbreviations

ATCC, American Type Culture Collection; IFO, Institute of Fermentation Osaka; LMG, Laboratorium voor Microbiologie, Gent; NRRL, ARS Culture Collection; NBRC, NITE Biological Resource Center; NMR, Nuclear Magnetic Resonance.
