# Supplementary material for: Acetan and Acetan-Like Polysaccharides: Genetics, Biosynthesis, Structure, and Viscoelasticity
Source: Polymers (Basel). 2021 Mar 7;13(5):815. doi: 10.3390/polym13050815 (PMC7961339; doi:10.3390/polym13050815)
Supplement: Supplementary file 1 [file polymers-13-00815-s001.zip › Supplementary figure 1.pdf]

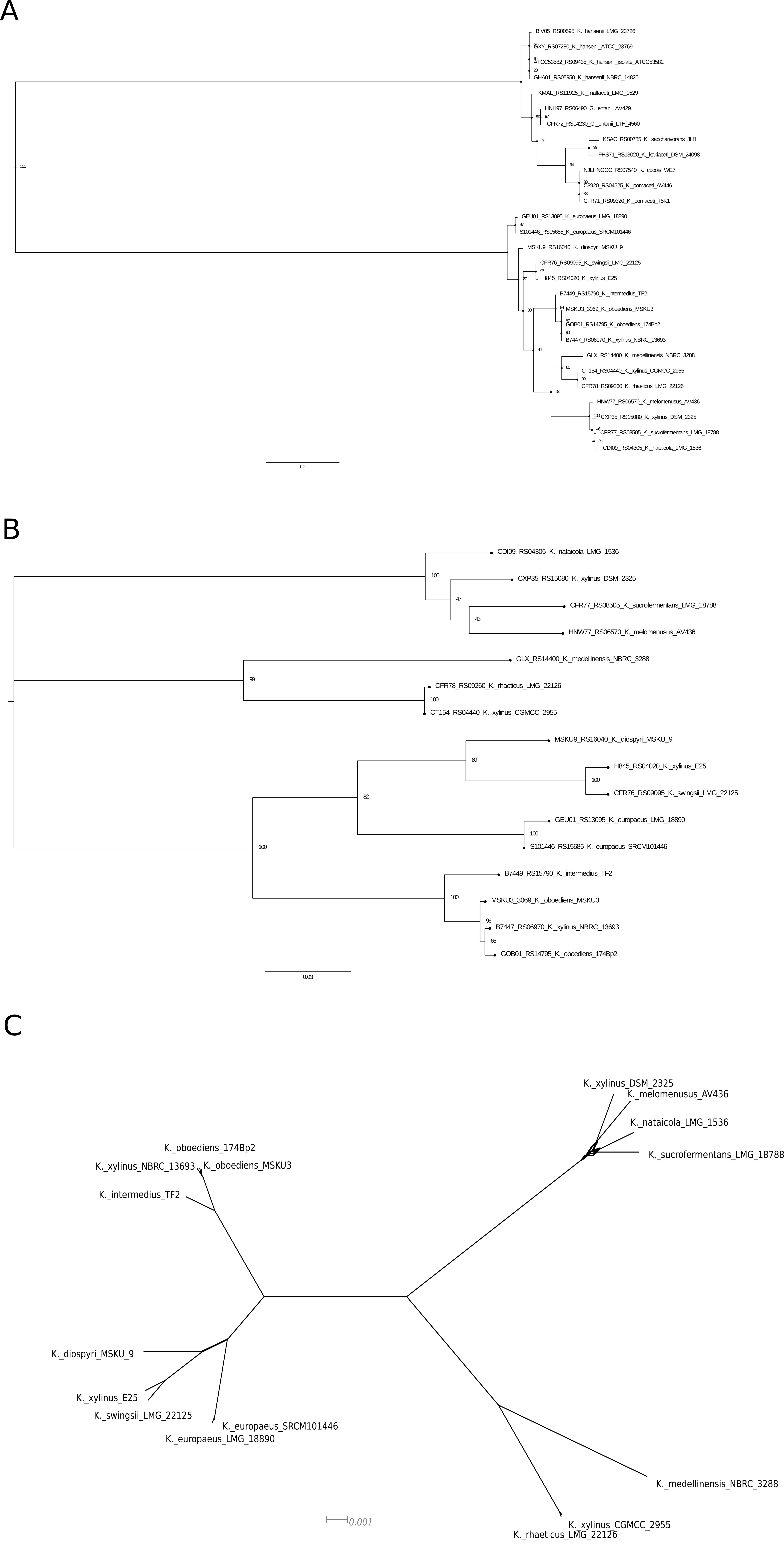

Supplementary figure 1. Divergence and putative horizontal gene transfer of acetan-like loci in *Komagataeibacter*. **A**: phylogeny of *Komagataeibacter* AceH as deduced by PhyML using LG substitution table and 1000 bootstrap replicates. The lower cluster corresponds to the archetypal *K. xylinus* E25 locus, while the upper groups the AceH belonging to the newly found locus. **B**: *aceH* gene phylogeny of *K. xylinus* E25 type acetan locus reconstructed using GTR model and 1000 bootstrap replicates. *K. xylinus* loci are dispersed among several well resolved clusters. **C:** Supernetwork analysis of 17 gene trees of the 16 *Komagataeibacter* strains harboring the archetypal *K. xylinus* locus. All the trees are essentially very similar implying the locus was transferred as a unit several times.
